# A Wasp Manipulates Neuronal Activity in the Sub-Esophageal Ganglion to Decrease the Drive for Walking in Its Cockroach Prey

**DOI:** 10.1371/journal.pone.0010019

**Published:** 2010-04-07

**Authors:** Ram Gal, Frederic Libersat

**Affiliations:** 1 Department of Life Sciences, Ben-Gurion University of the Negev, Be'er-Sheva, Israel; 2 Institut de Neurobiologie de la Méditerranée INSERM U901, Université de la Méditerranée, Parc Scientifique de Luminy, Marseille, France; Mount Sinai School of Medicine, United States of America

## Abstract

**Background:**

The parasitoid Jewel Wasp hunts cockroaches to serve as a live food supply for its offspring. The wasp stings the cockroach in the head and delivers a cocktail of neurotoxins directly inside the prey's cerebral ganglia. Although not paralyzed, the stung cockroach becomes a living yet docile ‘zombie’, incapable of self-initiating spontaneous or evoked walking. We show here that such neuro-chemical manipulation can be attributed to decreased neuronal activity in a small region of the cockroach cerebral nervous system, the sub-esophageal ganglion (SEG). A decrease in descending permissive inputs from this ganglion to thoracic central pattern generators decreases the propensity for walking-related behaviors.

**Methodology and Principal Findings:**

We have used behavioral, neuro-pharmacological and electrophysiological methods to show that: (1) Surgically removing the cockroach SEG prior to wasp stinging prolongs the duration of the sting 5-fold, suggesting that the wasp actively targets the SEG during the stinging sequence; (2) injecting a sodium channel blocker, procaine, into the SEG of non-stung cockroaches reversibly decreases spontaneous and evoked walking, suggesting that the SEG plays an important role in the up-regulation of locomotion; (3) artificial focal injection of crude milked venom into the SEG of non-stung cockroaches decreases spontaneous and evoked walking, as seen with naturally-stung cockroaches; and (4) spontaneous and evoked neuronal spiking activity in the SEG, recorded with an extracellular bipolar microelectrode, is markedly decreased in stung cockroaches versus non-stung controls.

**Conclusions and Significance:**

We have identified the neuronal substrate responsible for the venom-induced manipulation of the cockroach's drive for walking. Our data strongly support previous findings suggesting a critical and permissive role for the SEG in the regulation of locomotion in insects. By injecting a venom cocktail directly into the SEG, the parasitoid Jewel Wasp selectively manipulates the cockroach's motivation to initiate walking without interfering with other non-related behaviors.

## Introduction

Animals are not automatons that react identically every time they encounter the same stimulus [1]. Changes in the internal physiological state of an animal alter its responsiveness to stimuli and consequently affect its motivation to engage in a given behavior. Such processes and their underlying neuronal substrates have been the subject of extensive study for decades [2]–[4]. These efforts have undoubtedly benefited from studies on animals with relatively simple nervous systems, controlling stereotyped behaviors [1], [5]–[8].

Through millions of years of co-evolution, a few animal species have evolved unique strategies to control the motivation of their prey to engage in specific behaviors, thereby manipulating the prey in most exceptional ways [9]. One such example is the parasitoid Jewel Wasp (*Ampulex compressa*) which specifically depresses the drive of its prey to engage in locomotion [10]. The adult female wasp hunts cockroaches (*Periplaneta americana*) for use as a live food supply for its offspring. Since development of the wasp's larva requires feeding on live cockroaches for several days [11], the adult wasp does not kill or paralyze the cockroach prey but instead uses neurotoxins to selectively ‘hijack the free will’ of the prey. A cockroach stung by a Jewel Wasp first grooms itself excessively for 30 minutes, and then becomes hypokinetic for 3–7 days, during which time it loses the ability to self-initiate and maintain walking-related behaviors [12], [13]. The stung cockroach is not, however, paralyzed, allowing the wasp to grab its prey by the antenna and lead it to a nest, with the cockroach all the while following in a docile manner, much like a submissive dog on a leash (movie available online [10]). The wasp then lays an egg on the cockroach, seals the nest and leaves the docile prey inside. The wasp larva hatches two days later and feeds on the cockroach for another three days. The prey, although still alive throughout this process, does not put up a fight nor try to escape its tomb. The larva then pupates inside the cockroach's abdomen and hatches a month later as an adult, ready to continue its life cycle [11].

To render the cockroach hypokinetic, the wasp stings it twice, first in the thorax and then in the head [14]. The thoracic sting is brief and transiently paralyses the cockroach's front legs [15], [16] to facilitate the second and more precise sting into the head. The head sting is longer in duration and is responsible for the later behavioral alterations observed in stung cockroaches, i.e., excessive grooming, long-term hypokinesia and changes in the cockroach's metabolism designed to preserve nutrients for the developing larva [11]. To investigate where in the cockroach head does the wasp inject its venom, Haspel et al. [17] injected wasps with radiolabeled amino acids and traced the radioactive venom using autoradiography. In cockroaches stung in the head by such ‘hot’ wasps, venom was traced by and large inside the cockroach's cerebral ganglia, namely the supra-esophageal ganglion (SupEG) and sub-esophageal ganglion (SEG). Furthermore, Gal et al. [18] demonstrated that the wasp actively searches for, at least, the SupEG inside the cockroach's head capsule during the head sting. These findings suggest that the behavioral changes observed in stung cockroaches result from the neurotoxic effects of the venom on the SupEG, the SEG, or both. However, the role of each ganglion in inducing these behavioral changes is still unclear.

In insects, the cerebral ganglia are known to comprise the ‘higher-order’ neuronal centers implicated in modulating the thoracic Central Pattern Generators responsible for the spatio-temporal pattern of locomotion [19]–[25]. We have recently demonstrated that in stung hypokinetic cockroaches, the thoracic Central Pattern Generators are not directly affected by injected wasp venom. Rather, it is the drive to initiate and maintain walking-related behaviors that is selectively depressed in stung cockroaches, with minimal or no interference to other behaviors [10]. Since the SEG has been suggested to tonically up-regulate walking-related behaviors, while the SupEG appears to be generally inhibitory [24], we hypothesized in the present study that the SEG is the primary neuronal substrate responsible for the neuro-chemical manipulation of the cockroach's drive to initiate walking. To test this hypothesis directly, we have employed behavioral, neuro-pharmacological and electrophysiological tools to investigate whether specific modulation of neuronal activity in the SEG can account for the hypokinetic state of stung cockroaches.

## Results

### The wasp actively targets the cockroach's SEG during the head sting

**Figure 1:**
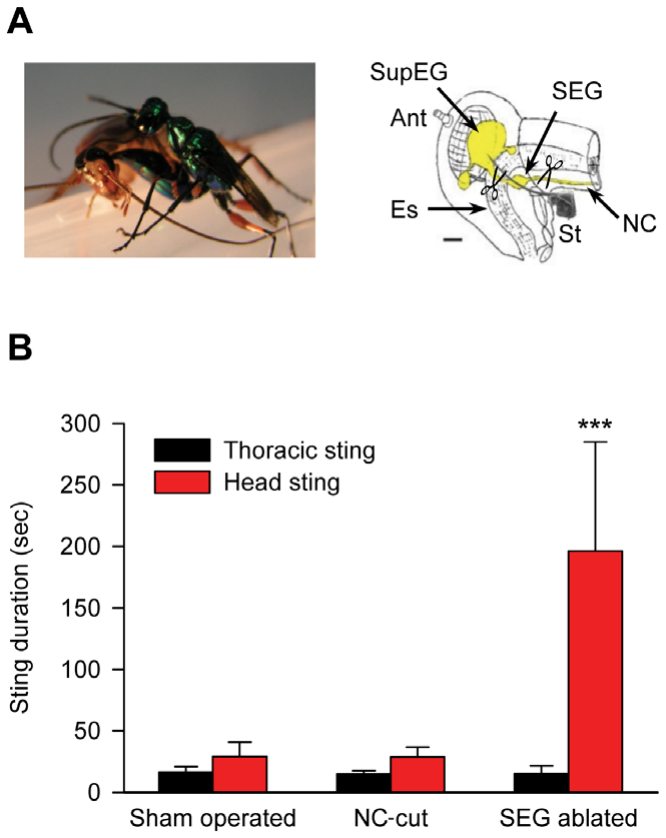
Effect of lesions in the cockroach CNS on the wasp's stinging duration. A. Left: A Jewel Wasp stinging a cockroach in the head. Right: Schematic lateral view of the cockroach head cavity (CNS is in yellow) and the wasp's stinger (St), shown as a scanning electron micrograph superimposed and drawn to scale, penetrating through the head cuticle to reach the cerebral ganglia (supra-esophageal ganglion, SupEG, and sub-esophageal ganglion, SEG). Locations of experimental CNS lesions are marked with scissors: In SEG-ablated cockroaches, the connectives rostral and caudal to the SEG were severed and the ganglion physically removed from the head cavity. In neck connectives-cut cockroaches, by contrast, only the neck connectives (NC) caudal to the SEG were severed, with the ganglion itself left intact. Es: esophagus. Ant: antenna. Scale bar: 0.5 mm. Modified from [17]. B. Wasp stinging behavior after specific experimental lesions of the cockroach's cerebral CNS. Cerebral lesions do not affect the duration of the first sting directed at the thorax (black bars). In contrast, physically removing the SEG from the cockroach head cavity prior to the sting (SEG ablated), but not cutting the cockroach neck connectives (NC-cut), significantly increases the duration of the second sting directed at the head (red bars). ***p<0.001, as compared with sham-operated and NC-cut cockroaches.

If the cockroach's SEG plays a crucial role in the venom-induced hypokinesia, then one would expect the wasp to actively target not only the SupEG [18] but also the SEG during the head sting. To test this hypothesis, we quantified the stinging behavior of wasps to which three groups of cockroaches were presented (n = 8 cockroaches in each group; Fig. 1A): (1) SEG-ablated cockroaches, namely cockroaches from which the SEG had been surgically removed prior to the sting; (2) Neck-connectives (NC)-cut cockroaches, in which the neck connectives between the thorax and the SEG were cut prior to the sting. These cockroaches, similar to SEG-ablated cockroaches, had no descending cerebral inputs reaching thoracic motor centers. Unlike SEG-ablated cockroaches, however, no neuronal tissue was physically removed from the head cavity of NC-cut cockroaches. Finally, as a control, wasps were also presented with (3) sham-operated cockroaches.

The duration of the first sting into the thorax was not significantly different for any group of cockroaches (SEG-ablated: 15±6 sec; NC-cut: 15±3 sec; sham-operated: 16±5 sec), demonstrating that cerebral lesions did not interfere with the wasp's motivation to sting or with its initial stinging behavior. Indeed, following the typical thoracic sting, the wasp readily pulled out its stinger and aimed it at the cockroach's head. The duration of the head sting, in marked contrast with the thoracic sting, was significantly longer when the wasps stung SEG-ablated cockroaches (196±88 sec, p<0.001), as compared with NC-cut or sham-operated cockroaches (39±8 sec and 39±12 sec, respectively) (Fig. 1B). There was no significant difference between stinging durations of NC-cut and sham-operated cockroaches (p = 0.941), showing that elimination of descending cerebral inputs to the thorax is not sufficient, by itself, to increase the head sting duration. Given these initial observations, *A. compressa* appears to actively search for the SEG inside the head capsule of its cockroach prey while delivering the head sting.

### Neuro-pharmacological inhibition of the SEG decreases walking in non-stung cockroaches

**Figure 2:**
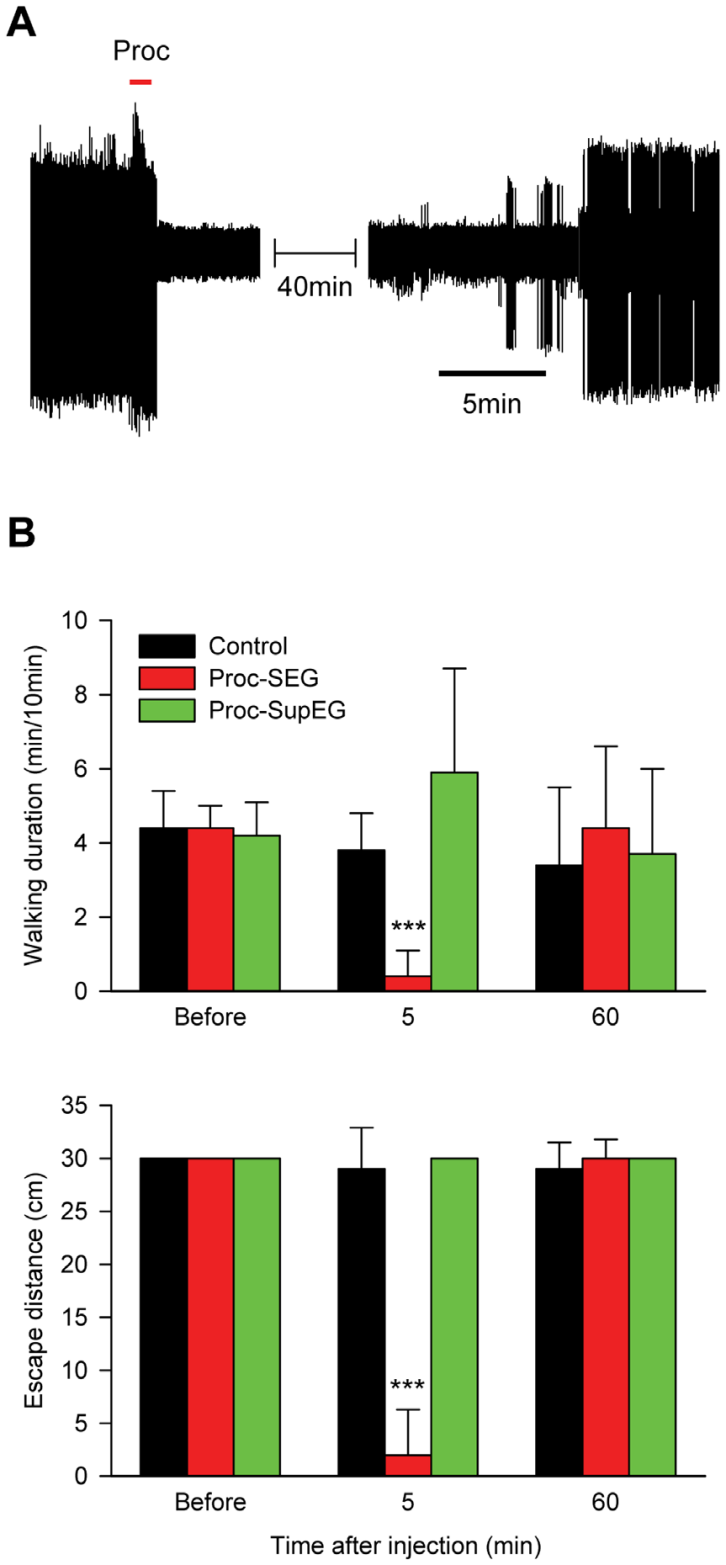
Procaine-induced inhibition of SEG neural activity depresses cockroach locomotion. A. Extracellular bipolar recording of spiking activity in the SEG of a non-stung cockroach. Transient (1 min, red bar) application of the sodium-channel blocker, procaine, completely and reversibly abolishes neuronal activity in the ganglion. B. Behavioral analysis of non-stung cockroaches injected with saline (black bars) or procaine into the SEG (red bars) or the SupEG (green bars). Spontaneous walking (top) and evoked escape responses (bottom) are reversibly suppressed by the inhibition of neuronal activity in the SEG but not of the SupEG. ***p<0.001, as compared with controls.

If the wasp's venom inhibits neuronal activity in the SEG, thereby depressing the cockroach's drive for walking, one would expect that focal neuro-pharmacological inactivation of SEG activity would similarly depress the drive for walking. To test this hypothesis, we used the reversible sodium-channel blocker, procaine, which has been shown to reversibly inhibit neuronal activity in the insect central nervous system [26], [27]. When we first tested the effect of procaine on a cerebral ganglion by applying it onto the SEG, the local anesthetic reversibly inhibited all neuronal activity in this ganglion (Fig. 2A). Accordingly, we injected procaine or saline (n = 14 for each group) focally into the SEG of non-stung cockroaches and assessed the behavioral outcome. As a control, we also assessed the behavior of cockroaches after focally injecting procaine into the SupEG.

Similar to a wasp's sting, procaine injection into the SEG dramatically depressed spontaneous and evoked locomotion (Fig. 2B). The anesthetic treatment significantly decreased spontaneous walking duration from 4.4±0.6 min to 0.4±0.7 min during a 10-min trial, with the distance of escape responses dropping from 30 cm before the injection to 2±4 cm afterwards. In contrast, procaine injected into the SupEG slightly increased spontaneous walking (4.2±0.9 min before injection and 5.9±2.8 min afterwards, p = 0.21) and did not affect the distance of escape responses (30 cm before injection and 30 cm afterwards). The inhibitory effects of procaine were specific to the anesthetic, as saline injected into any of the head ganglia did not significantly affect spontaneous or evoked walking (spontaneous walking duration: 4.4±1 min before injection and 3.8±1 min afterwards; escape distance: 30 cm before injection and 29±4 cm afterwards). Furthermore, the inhibitory effects of procaine were reversible, as 1 h after injection, no significant differences between procaine-injected and saline-injected cockroaches were noted in terms of walking duration (p = 0.245) or escape distance (p = 0.934). Thus, focal inhibition of neuronal activity in the SEG (but not in the SupEG) is sufficient to decrease the drive for walking in otherwise normally behaving cockroaches.

### Crude venom injected in the SEG depresses walking in non-stung cockroaches

Is the injection of wasp venom into the cockroach's SEG sufficient to decrease the drive for walking, in ways similar to procaine injection or a natural sting? To answer this question, we milked wasps and used a nano-injector to apply crude venom (or saline, in controls) directly and focally into the SEG or the SupEG of non-stung cockroaches (n = 5 in each group).

**Figure 3:**
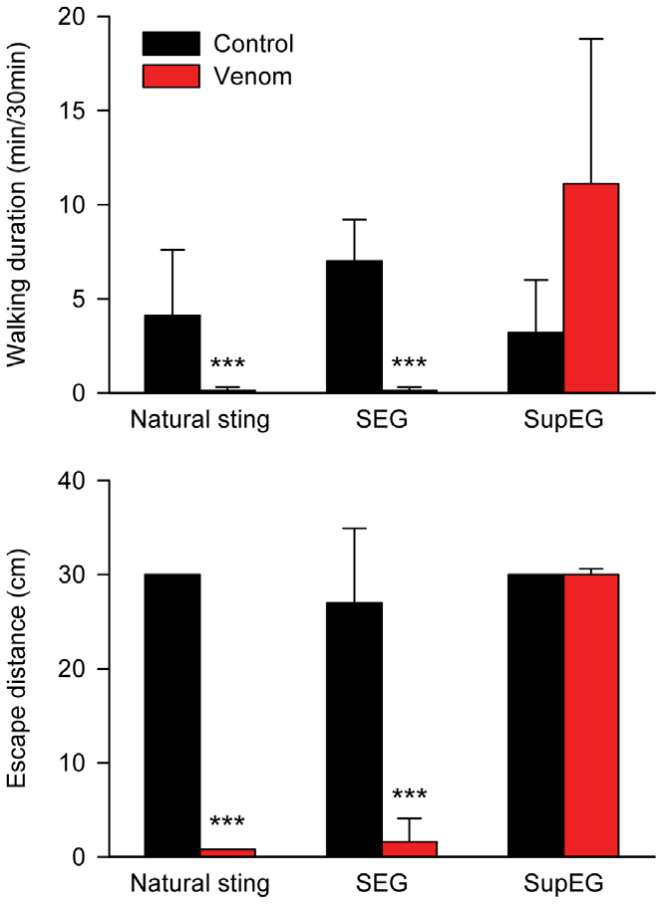
Behavioral analysis of stung and non-stung cockroaches injected with crude milked venom into different regions of the cerebral ganglia. Venom injected into the SEG, similar to a natural wasp sting, significantly depresses spontaneous walking (top) and escape responses (bottom). In contrast, venom injected into the SupEG has an opposite, albeit not significant effect. ***p<0.001 compared with the respective controls.

Crude venom injected into the SEG of non-stung cockroaches dramatically depressed walking (Fig. 3). Injected cockroaches spent very little time spontaneously exploring a novel arena (0.1±0.2 min, as compared with 7.0±2.2 min in controls; p<0.001) and failed to escape tactile stimuli (escape distance: 1.5±0.6 cm, as compared with 27±8 cm in controls; p<0.001). Overall, the behavior of SEG-injected cockroaches was highly similar to that of their naturally-stung counterparts (p = 0.19), who displayed a walking duration of 0.1±0.2 min and an escape distance of 0.8±0.1 cm. In contrast with venom injected into the SEG, venom injected into the SupEG tended to increase walking duration (11.1±7.1 min, as compared with 3.2±2.8 min in the appropriate control; p = 0.07) and did not impair escape responses (30±2.5 cm, as compared with 30±0.9 cm in the appropriate control). Thus, the presence of crude wasp venom in the SEG (but not in the SupEG) is sufficient to decrease the drive for walking in otherwise normally behaving cockroaches.

### Neuronal activity in the SEG is decreased in stung cockroaches

**Figure 4:**
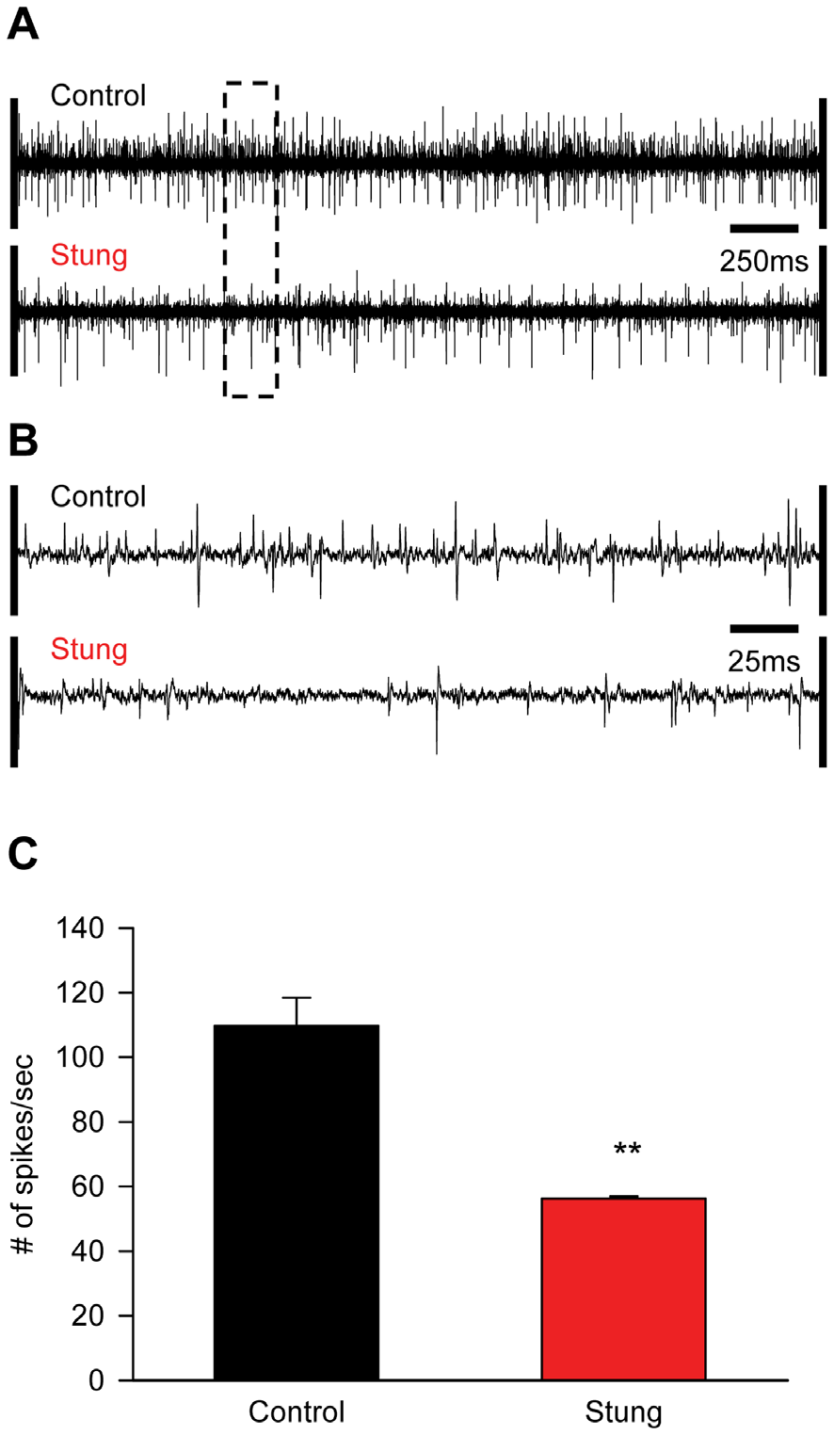
Spontaneous neural activity in the SEG of control and stung cockroaches. A, B. Extracellular bipolar microelectrode recordings of spontaneous spiking activity in the core of the SEG in one non-stung (‘control’) and one stung cockroach. The dashed region in A is enlarged in B. C. The spontaneous firing rate in the core of the SEG is significantly decreased in stung cockroaches. **p<0.05.

**Figure 5:**
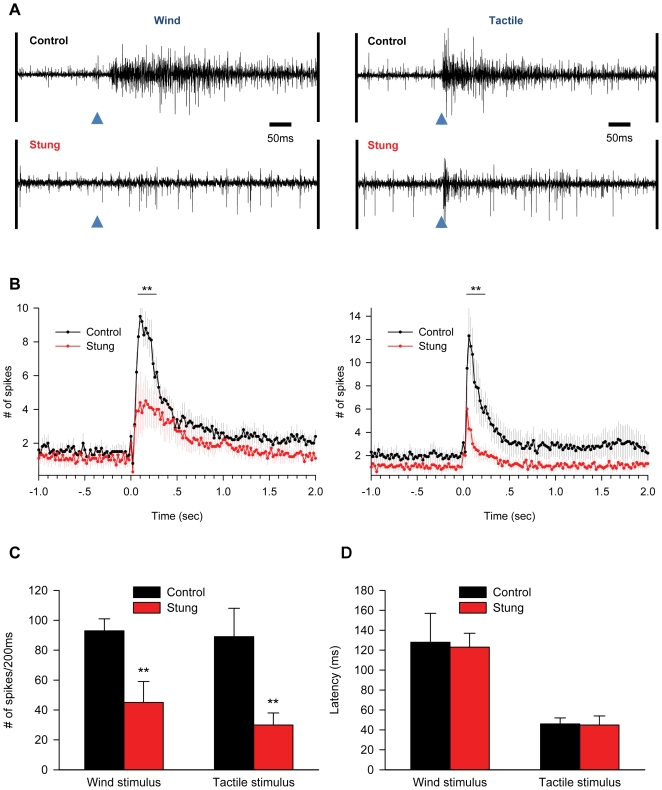
Evoked neural activity in the SEG of control and stung cockroaches. A. Extracellular bipolar microelectrode recordings of evoked activity in the core of the SEG in one non-stung (‘control’) and one stung cockroach (left: wind stimulus to the cerci; right: tactile stimulus to the antenna; arrows: stimulus onset). B. Peri-stimulus time histograms for wind (left) and tactile (right) stimuli. Each data point represents the mean (± SEM) number of spikes within a 20 ms time bin. The response to stimuli is decreased in stung cockroaches, especially during the first 200 ms after stimulus onset (represented by a bar above the histograms). C. The number of spikes during the first 200 ms immediately after the stimulus onset. Stung cockroaches show decreased responses to wind and tactile stimuli. **p<0.05. D. The latency between stimulus onset and maximal spiking response is similar in stung and control cockroaches.

To directly test whether the sting manipulates neuronal activity in the SEG, we compared the SEG activity of stung cockroaches to that of non-stung controls (n = 6 in each group). In these experiments, we used an extracellular bipolar electrode to record spontaneous and evoked spiking activity within the SEG (Fig. 4, 5). We focused our investigation on the central and middle (150–200 micrometers deep) portion of this small ganglion, corresponding to the natural venom injection site [17].

On average, spontaneous spiking activity in the core of the SEG was decreased two-fold in stung cockroaches, as compared with controls (56.3±0.6 spikes/sec and 109.7±8.7 spikes/sec, respectively; p<0.05) (Fig. 4). We further characterized this difference by applying wind stimuli to the cerci or tactile stimuli to the antenna (Fig. 5), both known to be poorly effective in eliciting escape in stung versus normal cockroaches [12], [14]. The number of stimulus-evoked spikes during the first 200 ms after stimulus application was significantly lower (p<0.05) in stung cockroaches, as compared with control cockroaches (wind stimuli: 45±14 spikes and 93±8 spikes, respectively; tactile stimuli: 30±8 spikes and 89±19 spikes, respectively) (Fig. 5B, C). Such a decrease in the SEG neuronal response is unlikely to be caused by changes in ascending sensory inputs, since the latency between stimulus onset and maximal neuronal response in the SEG was similar in stung and control cockroaches (wind stimuli: 123±14 ms and 128±29 ms, respectively; p = 0.878; tactile stimuli: 45±9 ms and 46±6 ms, respectively; p = 0.288) (Fig. 5D).

## Discussion

Cockroaches stung by the parasitoid Jewel Wasp (*A. compressa*), although not paralyzed, loose the ability to self-initiate locomotion for several days [18]. This deficit cannot be attributed to an overall sleep-like state for three main reasons. First, the deficit is highly specific, in that the threshold for initiation of other motor behaviors (such as righting, swimming, flight, etc.) is little affected [10]. Second, stung cockroaches do not assume a typical ‘quiescent’ position [28] and occasionally move their antennae in an exploratory manner. Third, when startled by a supra-threshold stimulus, stung cockroaches respond by jumping in place but do not perform the stereotypic subsequent run [14]. Thus, and since the sensory and motor systems *per se* are fully functional in stung cockroaches [12], [14], the wasp venom appears to specifically decrease the cockroach's drive for walking. The fact that the wasp injects its neurotoxic venom directly into the cockroach's cerebral ganglia to ‘hijack the cockroach's free will’ allows us to explore the neuronal substrate responsible for this unique behavioral manipulation.

Insect attention and arousal states, and their correlation with mammalian equivalents, have been thoroughly investigated during the last few years [29]–[33]. However, despite their obvious implications on the regulation of behavior, the neuronal underpinnings of motivation, or the drive to initiate specific motor behaviors, have received relatively little attention. As such, our present study aimed at employing the wasp-cockroach parasitic interaction to define the neural substrate responsible for the drive to initiate walking in insects. We show that the neuro-chemical manipulation performed by the wasp is achieved, at the least, by inhibition of neuronal activity in a small region of the cerebral ganglia of insects, namely the sub-esophageal ganglion (SEG). We propose this to be the case for the following reasons: (1) The wasp has been previously shown to use sensory feedback from its stinger to locate the SupEG within the cockroach head cavity when inflicting the head sting [18]. Hence, due to the anatomical location of the SEG on the trajectory to the SupEG, venom found in the SEG [17] could be, in principle, an incidental by-product of the sting prime target, the SupEG. In the present work, we show that this is not the case, since surgically removing the cockroach SEG prior to stinging, but not cutting the neck connectives without SEG removal, significantly increases the duration of the head sting. This suggests that during the head sting the wasp actively targets not only the SupEG, as previously reported [18], but also the SEG, unraveling the potential role of this ganglion in venom-induced hypokinesia; (2) *in vivo* pharmacological inhibition of SEG neuronal activity by procaine, a reversible sodium-channel blocker, reversibly decreases the propensity for spontaneous and evoked walking in non-stung cockroaches. Inhibition of the SupEG with procaine, in contrast, has little effect on spontaneous and evoked walking. These results are in agreement with previous studies (see [24] and references therein) which have suggested, using lesion experiments, that the SEG exerts a net tonic permissive effect on thoracic motor centers; (3) micro-injection of crude wasp venom into the SEG (but not into the SupEG) of non-stung cockroaches is sufficient to decrease the propensity for spontaneous and evoked walking, similar to what is seen in stung cockroaches; (4) spontaneous and evoked electrophysiological activity in the SEG is decreased in stung cockroaches, as compared with controls. Thus, our data unequivocally demonstrate the role of the SEG in the venom-induced inhibition of the drive for walking in cockroaches stung by *A. compressa*. To the best of our knowledge, these results provide the first direct evidence to support this long-standing hypothesis [14], [17].

Although the role of the SEG in the regulation of insect locomotion is, to date, still unclear, some previous evidence suggests that it exerts a permissive descending tonic effect on thoracic motor centers (see [24] and references therein). This is in contrast with the descending influence of the SupEG, where some neuronal structures (e.g. the Central Body Complex) seem to up-regulate, while others (e.g. the Mushroom Bodies) apparently down-regulate thoracic motor centers [22], [25], [34], [35]. In locusts, decision-making with respect to the selection and maintenance of walking, has also been examined using intracellular recordings of neurons in the SEG and the SupEG [36]. The spontaneous initiation of walking is accompanied by changes in the firing pattern of several SEG and SupEG descending interneurons. However, while SEG and SupEG interneurons both fire during walking, and are thus both involved in walking maintenance (see also [21], [37]), predominantly SEG interneurons fire during the preparatory phase of walking. This observation suggests a prime role for SEG neuronal circuits in determining the motivational level or ‘rest state’ of the animal [7] to engage into walking. Inhibition of SEG neuronal activity could therefore, in principle, decrease the propensity for expression of spontaneously initiated walking-related behaviors. Similarly, since the SEG sends permissive tonic inputs to thoracic pattern generators [24], inhibition of SEG activity could also depress walking in response to sensory stimuli, such as those used in the present study, although the stimuli used here do not involve a preparatory phase and the sequential recruitment of SEG and SupEG interneurons does not take place. Taken together, the results presented in this report strongly support such ‘rest state’ neuronal organization of higher motor control and suggest that selective inhibition of neuronal activity in the SEG is sufficient to decrease the drive for walking, without interfering with other behaviors or with the thoracic Central Pattern Generators directly.

The exact role of the SupEG in the venom-induced manipulation of the cockroach motor behavior remains, as yet, rather elusive. Several possibilities can be offered, such as a role in evoking the excessive grooming behavior seen in stung cockroaches [13], or importance for venom-induced changes in cockroach metabolism [11]. It is also possible that the SupEG, in concert with the SEG, plays a role in inducing certain aspects of venom-induced hypokinesia either directly, by affecting specific circuitries in this ganglion, or indirectly, by affecting ascending SEG interneurons which, in turn, modulate SupEG circuitries that control motor behavior. A direct effect of the venom on the SupEG apparently contradicts previous studies which showed that decerebrated insects (i.e., those without a SupEG) tend to walk uninhibitedly (see, for instance, [23], [24], [38]–[41]), suggesting a generally inhibitory effect of this ganglion on locomotion. However, it has also been shown that certain structures within the SupEG, and especially the Central Body Complex, affect some finer aspects of locomotion, including the frequency, duration and coordination of walking, turning behavior and obstacle climbing [19], [22], [23], [35], [42]–[46]. The venom could thus, in principle, specifically manipulate these SupEG structures, in addition to manipulating SEG activity, to further control the initiation of locomotory behavior in the cockroach prey.

The specific neurons within the SEG that are targeted by the venom to induce hypokinesia are currently under investigation. Prime candidates are neuromodulatory interneurons, in particular monoaminergic interneurons, descending from the SEG to thoracic motor centers and/or ascending from the SEG to the SupEG. One such population comprises the octopaminergic (OA) unpaired median neurons of the SEG, the axons of some of which innervate segmental ganglia, while others innervate major neuropiles in the SupEG [47]–[56]. Recently, activity in SEG-OA neurons of *Manduca* larvae has been correlated with fictive locomotion [57], further highlighting these neurons as major candidates for the venom-induced hypokinesia observed in cockroaches. Furthermore, we have recently shown that in stung cockroaches, the octopamine receptor agonist, chlordimeform, induces a significant increase in spontaneous walking when injected into the SupEG [53]. This suggests that the wasp's venom interferes with octopaminergic modulation of walking initiation in central structures of the cockroach SupEG.

To summarize, we have shown here that the wasp actively searches for the SEG of its host into which to inject its venom. Having previously shown that the wasp injects venom directly into the two cerebral ganglia [17] and that the venom's major effect is to decrease the drive for walking initiation [10], the novelty of the present study is the experimental verification that the wasp decreases the neuronal activity in the SEG to specifically down-regulate the drive for walking in its host. Given these facts, one can only wonder how, through millions of years of co-evolution, *Ampulex* has evolved this exquisite strategy to chemically control such ‘higher’ behavioral function in its host. By further identifying the neuronal basis of these parasite-induced alterations of host behavior, we hope to increase our understanding of the neurobiology of the selection and initiation of behaviors and the associated neural mechanisms underlying changes in responsiveness, both prime issues in the study of motivation.

## Materials and Methods

### Animals

*Ampulex compressa* Fabricius (Hymenoptera: Ampulicidae) wasps and *Periplaneta americana* cockroaches were reared in crowded colonies under laboratory conditions of 40–60% humidity, 30°C and a 12L:12D cycle. All animals were supplied with water and food (cat chow for cockroaches and honey for wasps) *ad libitum*. For stinging, an adult female wasp was introduced into a terrarium together with an adult male cockroach and allowed to afflict the full stinging sequence, namely a thoracic sting followed by a head sting. After stinging, the cockroach was immediately removed and isolated to prevent further manipulation by the wasp.

### Surgical procedures

#### General

Prior to all surgical procedures, cockroaches were anesthetized with carbon dioxide and immobilized with modeling clay on a wax platform. A staple-shaped insect pin was softly pressed against the neck to regulate hemolymph flow to the head during the procedure [24]. All cuticular incisions were allowed to seal by hemolymph coagulation. *Cockroach CNS lesions* (see Fig. 1A): To cut the neck connectives (NCs), a U-shaped incision was performed to open a small flap in the ventral head cuticle and the NCs were cut with fine micro-scissors. In SEG-ablated cockroaches, the circumesophageal connectives were subsequently severed and the SEG was physically removed from the head. In sham-operated control cockroaches, a flap was opened for 10 min with no interference with neuronal tissue. *Micro-injections:* A Nano-Volumetric Injector (NVI-570A/V, Medical Systems, Greenvale, NY) was used to deliver solutions directly into the cerebral ganglia (approximately 40 nl to the SEG or 100 nl to the SupEG). For SEG-injections, the cockroach was immobilized ventral side up, a flap was opened in the ventral head cuticle and the neck muscle gently moved aside to expose the ganglion. Injections were aimed and guided stereotactically, using ganglionic fiducials, to the middle and centre of the SEG, approximately 150–200 µm deep. For SupEG injections, the cockroach was immobilized dorsal side up and a small flap was opened between the compound eyes. Injections were directed between the two hemispheres of the protocerebrum, between the Mushroom Bodies and in the vicinity of the Central Body Complex, concomitant with the location of a natural wasp sting [17]. All injected solutions were added with an inert viable tracer (0.1% Janus Green) to allow tracing of the injection site *post mortem*.

### Pharmacology

Procaine was freshly prepared and dissolved to a concentration of 500 mg/ml in vehicle containing cockroach saline and 0.1% Janus Green. Venom was freshly milked from 10 wasps as described previously [16] and dissolved approximately 1∶10 in a vehicle containing 0.1% Janus Green, 10 mM HEPES buffer and 0.1 mM PMSF. Controls were injected with the respective vehicle alone.

### Behavioral assays

A detailed description of some of the assays performed here can be found in [24]. Briefly, all cockroach behavioral assays were performed on freely-moving cockroaches in an open-field arena (radius = 30 cm). *Spontaneous walking* was measured as the total duration of exploration of the arena during a 10-min (after procaine injections) or a 30-min (after venom injections) trial period. Walking episodes that occurred simultaneously with grooming were considered as walking episodes. *Escape responses* were measured as the distance the cockroach ran after receiving a tactile stimulus to the abdomen. The procedure was repeated three times with 1 min intervals and the results averaged for each cockroach and then pooled with the results of the entire group.

### Electrophysiology

#### Setup

Cockroaches were anesthetized with carbon dioxide, immobilized ventral side up after removing the legs and wings to stumps, and covered with a sheet of modeling clay to limit hemolymph loss and to prevent spontaneous righting or flight-like movements (see [24]). Next, the head was fixed to the recording platform with insect-pin staples and beeswax to prevent movement. The SEG was exposed by removing the mouthparts and neck muscle, and care was taken to minimize damage to the trachea. The ganglion was desheathed and perfused with isotonic cockroach saline [58] throughout the experiment. In electrophysiological experiments where procaine was used, we recorded ongoing activity for 30 min, then applied procaine (500 mg/ml in cockroach saline) onto the SEG for 1 min, and then washed the ganglion thoroughly with saline.

#### Stimulation

Cockroaches were allowed 15 min to recover from the surgical procedure and were then subjected to a stimulation protocol composed of wind and tactile stimuli (6–12 stimuli of each type) applied alternatively at 30 sec intervals. Wind stimuli were directed at the cerci in the tail-to-head direction with a custom-built wind generator [59] which delivered wind puffs of roughly 150 ms in duration. To apply tactile stimuli to the antenna, we first prevented spontaneous antennal movements by holding the antenna in place using a staple-shaped insect pin. The pin was pushed into the wax-coated recording platform so that it very lightly pressed the base of the antenna against the platform. This confined the antennal flagellum to movements of <0.1 cm in the lateral plane and <0.1 cm in the dorso-ventral plane. The stimuli were presented to the middle of the antennal flagellum, approximately 3 cm from the scape, using a steel rod which briefly (∼60 cm/sec) deflected the antenna 1.5 cm medio-laterally. Such a stimulus induced a lateral bending of the base of the antenna against the confining pin, which we empirically determined to evoke the most spiking activity in the core of the SEG. Furthermore, the effect of this stimulus approximates the natural condition [60], where the wasp bends and then cuts the cockroach antennae after the sting. While such bending of the antenna reliably evokes a rapid escape response in normal cockroaches, it fails to evoke such a response in stung cockroaches. *Recording*: We recorded spiking activity from the center and middle of the SEG, 150–180 µm deep in the ganglion, with an extracellular bipolar tungsten microelectrode (1 MΩ, 1 µm tip diameter and 125 µm tip spacing; World Precision Instruments, Sarasota, FL). We chose this region for three main reasons: First, during a sting, the wasp injects its venom mainly into the middle and center of the ganglion, in and around the middle neuromere [17]. Second, in control cockroaches, we found this region to be the most spontaneously and reliably active, as well as demonstrating the widest variety of ongoing spike shapes and sizes. Third, we found this region to be the most responsive to tactile and wind stimuli, as described above. The electrode was guided stereotactically using a finely scaled micromanipulator and trachea as ganglionic fiducials. In preliminary experiments, the tip of the electrode was dipped in a solution of fluorescent dye (DiI, Biotium, Hayward, CA) prior to recording, and the ganglion observed as a whole mount *post mortem* to evaluate the exact recording site.

#### Analysis

We analyzed spiking activity one second before a stimulus (‘spontaneous activity’) and two seconds afterwards (‘evoked activity’). For each cockroach and for each stimulus type, such 3-sec recordings were divided into 20 ms time bins and the total number of spikes in each bin was counted and averaged across repeats to yield the individual average response of a cockroach. This response was then averaged across different cockroaches to produce the pooled peri-stimulus time histogram presented in Fig. 5B. Stimulus-response latency was calculated as the time between the onset of the stimulus and the peak neuronal response, defined as the maximum number of spikes within a 20 ms time bin. The stability of the recording quality throughout the experiment was controlled by calculating the percent change in wind-evoked spikes between the first and last stimuli applied during the stimulation protocol. In this study, we only included experiments in which this change did not exceed 20%, which we consider as acceptable variability for extracellular *in vivo* recordings. Spikes were acquired, sorted and analyzed with Spike2 data acquisition software (CED, Cambridge, UK) on a personal computer. All pooled electrophysiological data are presented as mean ± SEM.

### Statistical analysis

We used Student's t-test to analyze normally distributed data or the Mann-Whitney Rank Sum Test for non-normally distributed data. Except for the electrophysiological data described above, all data in this work are presented as mean ± standard deviation, with n representing the number of animals considered.
